# Supplementary material for: Are carbon emissions trading and green financial instruments synergistic? -Comprehensive quantitative research based on content analysis
Source: PLoS One. 2024 Mar 7;19(3):e0298601. doi: 10.1371/journal.pone.0298601 (PMC10919601; doi:10.1371/journal.pone.0298601)
Supplement: S1 Data — (ZIP) [file pone.0298601.s001.zip › Data/地级市Z_F工作报告.zip!/╔╜╢1⁄2/╡┬╓▌╩╨/└┤╫╘╣1⁄2╓┌║┼í╛┬φ┐╦╩2╛▌═°í┐_2014.docx]

各位代表：

现在，我代表市人民政府向大会报告工作，请予审议，并请市政协委员和其他列席会议的同志提出意见。

2013年主要工作回顾

过去的一年，市政府在中共德州市委的坚强领导下，在市人大、市政协及社会各界的监督支持下，团结依靠全市人民，深入贯彻党的十八大精神，以科学发展观为指导，牢牢把握主题主线主基调，紧紧围绕既定目标，科学运筹谋划，大胆探索创新，积极推进落实，全面完成市十七届人大二次会议确定的年度任务。

(一)经济平稳较快增长。面对宏观经济下行压力，抓转调促升级，增总量上水平，主要经济指标增幅位居全省前列。预计实现地区生产总值2460亿元，同比增长11.2%；公共财政预算收入150亿元，增长15.7%；全社会固定资产投资1680亿元，增长21%；实际利用外资2.1亿美元，完成进出口总额34亿美元，增长24.9%；城镇居民人均可支配收入24684元、农民人均纯收入10850元，分别增长10%和13%。经济结构不断优化，三产占比提高1.1个百分点；发展质量明显提高，税收占公共财政预算收入比重达到77.9%，提高4.7个百分点；县域经济发展活力进一步增强，2个县(区)公共财政预算收入过20亿元，7个县(市、区)主体四税收入增幅超过25%，5个县(市、区)提前完成县域经济“三年倍增”计划。现代农业发展步伐加快，粮食生产实现“十一连增”，总产达到166.5亿斤，设施农业、规模化养殖、农机作业率均居全省前列。工业经济快速增长，规模以上工业实现增加值1450亿元、利税848亿元、利润487亿元，分别增长14%、16%和16%；实施技改项目620个，完成投资450亿元，增长20%。服务业发展势头良好，实现社会消费品零售总额990亿元，增长13.3%；欧乐堡梦幻城、四女寺景区、黄河故道温泉度假村、渤海金山文化城等一批文化旅游综合体加快建设。

(二)产业发展后劲增强。招商引资纵深推进，组织开展百日会战，深入对接央企强企，成功举办北京、上海、天津、广州合作恳谈会，组团赴美国、香港、台湾等地招商，德州经济技术开发区与台湾中部科学园缔结姊妹园区，全市签约项目759个，美国威讯半导体、联孚新能源汽车、沈阳机床产业园、济铁物流园等542个重大招商项目开工建设。培大扶强有序展开，重点培植的60家骨干企业、55家科技成长型企业加速扩张，有12家企业主营业务收入过50亿元、3家过100亿元。鑫华润、格瑞德、齿轮公司等8家企业启动退城入园建设，永锋、恒源、金能、景津等骨干企业和奇威特、百多安、迈特力重机等科技成长型企业发展势头强劲。重大项目建设进展顺利，105个市级重点项目完成投资609亿元，华鲁恒升、桦超化工、玲珑轮胎、力驰科技、和胜电器等54个产业项目竣工投产，形成一批新的财源基础。科技驱动能力增强，新增国家级高新技术企业3家、省级以上科技创新平台15家、博士后科研工作站3个，高新技术产业产值占规模以上工业总产值的比重达到25.4%。金融体系建设步伐加快，金融生态环境不断优化，成功举办第三届资本交易大会，引进设立民生银行、青岛银行等各类金融机构63家，新增贷款335亿元，新增间接上市和挂牌企业26家，直接融资202亿元；采用多元化融资手段，市本级筹集重大基础设施建设资金28.5亿元，确保了年度重点建设项目的资金需求。

(三)城乡建设成效明显。中心城区建设取得突破性进展，共实施6大类68个城建项目，完成投资208亿元。 “两河四岸”滨河路基本建成，形成了纵贯城市南北的交通大动脉。新建改造水、气、热管网174公里，增强了城市保障能力。红星美凯龙、万达广场、唐人中心、蓝天百脑汇、绿城长河等城市综合体建设进度加快，袁桥、沙王、项目东区北部和南部等已开工大社区完成投资36亿元。城市数字化管控、精细化管理水平明显提升。城镇综合承载能力进一步增强，各县(市)组织实施了一大批公共建设项目，产业园区扩容提质，城区面貌明显改善，生态环境不断优化，县城在县域经济发展中的核心地位和拉动作用更加突出；12个示范镇启动建设项目135个，完成投资8.5亿元。 “两区同建”稳步实施，新建续建规模以上农村社区314个，建设1000亩以上农业产业园区373个，被国家农发行列为支持农村新型城镇化建设试验区，土地节约集约利用改革试验列入省部重点支持地区。大路网建设顺利推进，德大、邯济铁路和济乐、德商高速加快实施，石济客运专线开工，新建改造农村公路800公里，新增4个省村级公路网化示范县。大绿化工程成果丰硕，集中力量实施城市高标准景观绿化和乡村大面积生态绿化，森林覆盖率达到33%，成功创建国家园林城市。大水网构建成效明显，南水北调主体工程和大屯水库向城区供水工程竣工，实现黄河水、长江水双水源保障；农村饮水安全工程全面完成，成为全国第一个实现整建制城乡供水一体化的地级市；完成德惠新河清淤治理，5条主要河流水质基本达到地表水Ⅴ类标准；城乡水源地水质达标率100%，被评为“全国节水型社会建设示范区”。生态环境持续改善，海河迎查扎实开展，开工建设了污水处理、污泥处置、人工湿地等一批重点工程；实施蓝天行动计划，加大对燃煤企业、建筑工地等重点污染点源的治理力度，启动“黄标车”淘汰工作；强力推进节能减排，淘汰落后生产线20条、设备895台(套)，完成省下达的单位GDP降耗和主要污染物总量减排年度任务。

(四)社会事业扎实推进。全年重点民生领域财政支出165.6亿元，增长13.8%，占财政总支出的比重达到61.9%。民生实事完成年度任务，一批热点难点问题得到有效解决。城乡教育全面发展，开工建设农村中小学校舍标准化项目150个，新建改造幼儿园330所，补充教师2110人，实施德州学院东扩和德州二中规范化改造项目，我市被省政府确定为省级职业教育创新发展试验区，各级各类学校办学条件和教学质量进一步提升。公共卫生工作扎实开展，围绕解决看病难、看病贵的问题，大力推进医疗服务标准化和县级公立医院综合改革试点，174家医疗机构实行“先诊疗、后结算”模式；全面提高新农合筹资标准和报销比例，大病患者住院费用实际补偿比例由65%提高到85%。人口计生部门免费为15.9万人进行孕前优生健康检查。积极促进就业创业，新增城镇就业7.3万人，农村劳动力转移就业10.5万人。社会保障扩面提标，受益群众人数大幅增加，社保基金突破60亿元大关，社保待遇平均增长20.2%；企业退休人员月均增加养老金200元，实现“九连涨”；城市低保标准平均提高到每人每月332元，农村低保标准提高到每人每年2000元，五保分散和集中供养标准分别提高到每人每年2400元和3500元。开工建设保障性住房15966套，基本建成11932套，更多低收入家庭住房条件得到改善。公共文化服务体系建设取得新成效，市博物馆、档案馆新馆、德州大剧院全面启用，圆满完成“十艺节”承办任务；京杭大运河德州段通过世界遗产组织现场评估。全民健身活动丰富多彩，成功举办国际自行车邀请赛、肥仔艇大赛等一批国家级赛事。社会管理创新扎实有效，信访案件办结率、群众满意率同步提升。社会大局持续稳定，立体化治安防控体系进一步完善，“警务社区六进”成为全省典型。安全生产形势平稳，未发生重特大安全生产事故。食品药品、工商、质监管理体制改革顺利进行。双拥共建、民族宗教、妇女儿童、老龄助残、慈善、红十字会等社会事业持续发展，防震减灾、应急管理、司法仲裁、外事侨务、人防、史志、打私等工作取得新成效。

(五)政府自身建设不断加强。重视发挥政策引领作用，调整招商引资、企业培植等重大导向性政策，制定重点工程专项规划和教育、医疗等公共资源布局指导性意见。加强政府规范化管理，修订完善了 《市政府工作规则》，对规划建设、土地管理、财政预算、投资评审等重大事项，优化制度设计，严格程序要求，政府运行更加规范。大力简政放权，取消行政审批项目109项，下放34项，保留88项，精简幅度达到68%。强化市政务服务中心职能，办理服务事项150万件，审批材料、环节和时间压减三分之一。规范市级公共资源交易平台，完成交易项目483件、成交金额154亿元。加大政务公开力度，推进依法行政，自觉接受人大、政协监督，广泛听取各方面意见，共办理人大代表建议和政协提案 764件，办结率 100%。“12345”市民热线受理有效诉求56.9万件，综合办结率99.1%，办理结果回访满意率87.5%。认真落实改进工作作风、密切联系群众各项规定，深入推进惩治和预防腐败体系建设，行政监察、审计监督工作不断强化，保持了为民、务实、清廉的政府形象。

各位代表、同志们，2013年是不平凡的一年。我们为了更好地履职尽责，不负重托，一年来，我们始终注重超前谋划，增强工作前瞻性。坚持用长远眼光看待问题，以战略思维谋划工作，准确研判发展趋势，利用好政策，防控好风险，牢牢把握工作主动权。一年来，我们始终注重继承创新，增强工作连续性。围绕既定部署，大胆创新方法，积极创造条件，确保重点工作有序推进、任务目标全面完成。一年来，我们始终注重一线落实，增强工作实效性。推行一线工作法，大力倡导一线办公，既抓任务部署、又抓过程管控，既重结果、又看过程，切实提高政府执行力。一年来，我们始终注重完善制度，增强工作规范性。按规矩办事，按程序运作，使各项工作、各个环节有规可依、有章可循，全力推进政府工作的规范化、制度化、科学化。

各位代表，德州能有今天的发展局面，是上级党委、政府和市委正确领导的结果，是市人大、市政协和社会各界监督支持的结果，是全市人民团结进取、厚德包容、创新图强的结果。在此，我代表市政府，向全市人民，向各位人大代表、政协委员、离退休老同志，向各民主党派、工商联、人民团体和各界人士，向驻德部队官兵，向中央、省驻德单位，向关心支持德州发展的海内外朋友，致以崇高的敬意并表示衷心的感谢!

各位代表，我们清醒地认识到，与发展新要求、群众新期待相比，还存在一些问题和不足。主要是：产业链条短、关联度低，龙头企业拉动力弱，整体效益水平不够高；环境资源约束压力越来越大，集约发展、节能减排任务艰巨；教育、医疗等公共资源的供给总量和空间布局，与城乡发展和群众需求不相适应；政府深化改革、转变职能还有大量工作要做，各层面执行力和服务效能需要进一步提升。我们一定高度重视，认真研究解决。

2014年总体安排和重点工作

各位代表，今年是全面深化改革、加快科学发展的重要一年。根据上级要求和市委决策部署，政府工作的总体思路是：深入贯彻落实党的十八大和十八届三中全会精神，坚持稳中求进、进中求好、好中求快，把改革创新贯穿经济社会发展各个领域、各个环节，积极推动工作指导重大转变，努力提高经济发展质量和效益，强力突破产业发展、城市建设、城乡统筹、民生改善、生态建设五大重点，促进经济持续健康发展、社会和谐稳定，推动幸福德州建设取得新进展。

全市经济社会发展的主要预期目标是：地区生产总值增长10.5%左右，公共财政预算收入增长13%左右，固定资产投资增长18%，社会消费品零售总额增长13%，外贸进出口增长6%，利用外资稳定增长，城镇居民人均可支配收入和农民人均纯收入分别增长11%、13%，城镇化率达到49%，城镇登记失业率控制在3.5%以内，居民消费价格涨幅控制在3.5%左右，人口自然增长率控制在10.09‰以内，全面完成省下达的年度节能减排任务。

上述目标是根据中央和省里的总体要求，综合考虑质量效益导向、远近目标衔接、科学跨越需要等多方面因素，经过反复论证和审慎决策确定的，既积极稳妥，又留有余地。实现这些目标，德州有优势、有基础、有条件。 “一圈一带”、首都经济圈、环渤海经济圈、黄三角高效生态经济区五大战略带来的政策红利，高铁优势和生态科技城形成的平台效应，以及招商引资、抓大培强积累的坚实工作基础，都是德州发展的有利条件和希望所在。利用好这些条件，办好德州的事业，当前和今后一个时期，我们重点把握四条原则：一是产业发展突出导向性。根据我市资源条件、产业基础及未来趋势，市委、市政府经过充分论证，提出重点培植十大产业集群。主要包括农副产品(食品)深加工、装备制造、绿色化工、纺织服装四大传统优势产业集群，新能源与节能环保、生物技术、新材料、电子信息四大新兴战略产业集群，以及商贸科技金融、文化体育旅游两大现代服务业集群。在整体布局上，加强规划引领，高水平编制各产业发展专项规划，集中建设30多个重点产业基地。在发展模式上，突出抓好专业园区、主导产业、龙头企业三大载体，力促产业向规模化、高端化、集群化发展。在推进路径上，坚持非均衡配置资源，集中有限要素集约投放，推动重点产业、重点企业率先崛起。二是城乡建设把握阶段性。中心城区经过近几年的大投入、大建设，拉框架、打基础阶段基本完成，今后的发展重点将全面转向强配套、提功能，以教育、医疗等公共资源优化配置引导老城区有机疏散、新城区协调发展，快速提升承载力、影响力，建设鲁西北、冀东南区域性中心城市。县城以扩大城市规模和经济容量为目标，重点增强经济功能和服务功能，提高产业聚集度和人口吸纳力。乡镇以提升公共服务能力为重点，完善就业、就学、就医条件，逐步健全“四供两排”保障体系，建设一批有产业特色、有完备功能、有历史记忆的美丽城镇。农村社区以改善生产生活条件为重点，不断健全交通、供电、供水、通讯等基础设施和基本公共服务体系，使农民群众逐步过上现代化生活。三是民生事业注重持续性。改善民生没有终点站，只有新起点。按照“守住底线、突出重点、完善制度、引导舆论”的思路，加快基本公共服务体系建设，建立健全长效机制。坚持问题导向，把关系群众切身利益的热点难点问题作为工作重点，集中力量突破薄弱环节，真正把民生实事办到群众心坎上。根据需要与可能，分清轻重缓急，实行多元化投入，普惠政策全覆盖，困难特例兜住底，织好社会救助安全网。四是改革创新体现统领性。坚持以改革统领经济社会发展全局，注重顶层设计，把握节奏路径，正确、准确、协调、有序推进改革，确保用改革的思路和办法破解发展难题，引导发展方向，增强发展活力。重点是划清政府和市场的边界，政府应该办的，要办实办成办好；该由市场机制运作的，政府不包办，发挥好市场配置资源的决定性作用。处理好政府和社会的关系，加快实施政社分开，创新社会治理模式，发挥社会组织作用，实现政府治理和社会自我调节、居民自治良性互动。政府自身改革要体现简政放权、依法行政理念，推行权力清单，降低行政成本，实现行政权力公开透明运行。

围绕实现今年总目标，重点抓好以下六个方面工作：

(一)坚定不移地培植十大产业集群。总的目标是，用3至5年的时间，形成百亿级企业、千亿级产业、万亿级规模，即：每个产业都有主营业务收入过百亿元的龙头企业，每个产业主营业务收入超过千亿元，十大产业集群总体规模超过万亿元，推动德州实体经济总量翻番、财政贡献能力倍增。

继续打好招商引资攻坚战。突出抓好产业链招商，重点引进行业龙头、领军企业和重大配套项目。瞄准京津冀、长三角、珠三角和港台等重点区域，走出去、请进来相结合，精心举办专题招商推介活动，积极吸引和承接产业转移。紧盯央企、强企、上市公司，加快引进央企国资、优质民资、高端外资，抓好美国科泰克、奥特莱斯、广药集团等重大项目的跟踪落实，争取尽快落地。力争全年引进落地过10亿元项目中心城区20个以上、每个县(市)5个以上。

倾力培植产业龙头。坚持分类指导、综合施策，确保有基础、成规模的支撑性企业加速升级扩张，有潜力、劲头足的成长型企业快速膨胀。全面落实60家骨干企业和55家科技成长型企业培植计划，力争新增主营业务收入过100亿元的企业3家。大力促进装备升级改造，实施388个工业重点项目。抓好企业素质提升工程，积极推进股份制改造，加强企业家培训。同时，加快中小微企业发展，全面落实各项优惠政策，完善融资担保、创业辅导、信息咨询服务体系，新增“四上”企业1700家，推动创业创新创优群体快速膨胀。

加快打造产业基地。搞好十大产业集群研究分析，立足发展基础和特色优势，依托现有产业园区，抓好基地规划，推动企业集中布局、要素集中投放，迅速扩大规模，形成集聚效应。中心城区按照产城融合的理念，加快发展新能源与节能环保、电子信息、装备制造、绿色化工等专业园区，科学布局商贸科技金融、文化体育旅游等现代服务业组团。各县(市、区)根据自身条件，重点规划建设2—3处规模大、聚集度高的产业基地。积极发展飞地经济，加快台湾科学园、上海张江高科示范园、上海浦发科技创新产业园、北京中关村(齐河)产业园建设。大力推进外经外贸发展方式转变，积极争取设立综合保税区，加快青岛保税港区(德州)功能园区建设，推动有条件的外向型企业建立保税仓库，提高开放型经济水平。搞好省级开发区发展定位，抓好规划和基础设施建设，提高园区经济占县域经济的比重。依托省会城市群经济圈，支持禹城、齐河、临邑建立产业协作区。争取禹城高新区晋升国家高新区、齐河经济开发区晋升国家级开发区。

全面强化科技金融保障。把科技金融的融汇创新作为产业集群扩容升级的主引擎。加快应用技术研究院规划建设，搭建高端创新公共研发平台。整合科技资源，组建产业技术创新联盟。引导每个县(市、区)建成1处科技创新创业园、1处高新技术产业园。引进高端人才和科研团队，推动企业与高校院所开展产学研合作。规划建设金融商务区，提升资本交易大会规格层次，加快集聚各类金融资源，建设鲁冀边界区域性金融中心。新设立金融机构60家以上，其中引进商业银行3家以上，推进农村信用社改革，实现村镇银行县域全覆盖。继续整治金融生态环境，不良贷款率降到3%以下。拓宽融资渠道，加快推进企业上市挂牌步伐，确保全年新增贷款385亿元，直接融资220亿元，满足产业发展资金需求。

(二)坚定不移地打造区域性中心城市。建设区域性中心城市是省 “一圈一带”发展规划赋予德州的功能定位，也是全市人民的殷切期盼。大的城市构架是“一心四极”，当前主攻方向是中心城区。综合研究论证，中心城区的建设思路是“优化布局、提升功能，突出重点、打造亮点，服务民生、改善环境，量入为出、积极作为”。总的任务安排是，以生态科技城、外滩商务带等重点片区为支撑，以重点路段和重点部位为基础，以重点工程为载体，坚持以市场运作和政府投资相结合、以市场运作为主的原则，今年计划实施6大类70余项建设项目，概算投资约170亿元。

以大规划引领建设。继续深化城市总体规划，统筹布局生产、生活、生态三个空间，有序融合老城、新城、生态科技城三个组团，加快老城改造、新城提质、生态科技城提速，推动德陵一体化发展。科学编制产业园区、综合交通、基础设施、绿地水系、教育卫生等专项规划，抓紧完成高铁商务区、外滩商务带、城隍庙片区控制性规划，完善老工业区、老商业区、老居住区改造提升规划。加强建设项目修建性详细规划编制管理，明确规划条件，严格技术标准，强化景观设计，优化城市色彩，确保每一个建设项目都符合城市发展要求。

以大片区支撑全局。坚持以大片区拉动大建设、以大产业充实大平台，着力规划建设生态科技城、外滩商务带和南部生态片区三大支撑点，打造城市的标志性区域。生态科技城，规划面积150平方公里，以京沪高铁、石济客运专线为轴线，西起减河、东至东外环、北至北外环、南至南外环，有机整合高铁新区、项目东区和马颊河生态岛，建成科技创新先行区、现代产业聚集区、对外开放引领区、生态建设示范区和科学发展新的增长极。空间布局上分为“一核、两区、多园”，包括十大功能板块。 “一核”，重点以高铁站为核心，打造东西两大高铁广场，建设高端商务区，规划布局科技创新、商业服务、研发总部、金融投资四大板块。 “两区”，建设减河、马颊河生态岛两大生态功能区，充分发挥河流水系自然资源优势，规划布局优质教育、生态居住、医疗养老、旅游度假四大板块。 “多园”，包括高铁新区产业园区、项目东区和抬头寺部分区域，重点布局新型工业和现代物流两大板块，打造电子信息、装备制造、新能源与节能环保、现代物流等高端高质高效产业园。德州外滩商务带，南起新河路、北至大学路，全长4.7公里，依托岔河水系，加快万达广场、唐人中心两大城市综合体建设，启动周边片区总体改善升级，高标准实施滨河路景观绿化，设计建设中央公园，有机串联长河公园景区，把岔河两岸打造成现代都市滨河商务休闲带。南部生态片区，规划面积18平方公里，重点布局文化动漫、生态旅游、都市农业等绿色低碳产业，加快华嬉园和市动植物园建设进度，做好太阳湖景区前期工作。今后逐步向东延展至仙人湖、丁东水库“两湖”水系，与马颊河生态岛连为一体，形成城市南部生态屏障。

以大工程提升功能。重点实施道路街巷、公共设施、城市综合体和大社区等六大类工程。完成“两河四岸”道路后续工程建设，全线贯通大外环，新建改造三八西路、崇德九大道等主干道路，改造提升57条背街小巷。完成高铁综合客运站主体工程，启动汽车北站、快速客运站建设。新建改造供热、供水、供气、排水管网72公里，第四净水厂、高铁新区西区污水处理厂、餐厨垃圾处理厂等工程建成启用。加快推进中商产业智慧总部基地、芭蕾雨奥特莱斯商务综合体等重点工程开工建设，抓紧在生态科技城打造一批辐射力强、业态先进的商务组团。完成袁桥、项目东区南部和北部3个大社区建设，加快沙王、林庄等大社区建设进度，启动邹李等16个城中村、棚户区改造工程，加快“三无”小区改造，改善市民居住条件。

以大整治改善环境。结合文明城市创建，实施四大工程，改善市容市貌，提升城市品位。实施治堵保畅工程，深入开展“平安行·你我他”道路交通安全整治活动，提高道路通行能力。实施治违打非工程，拆除违章建筑，规范建设行为。实施治脏清污工程，清理垃圾、裸土、污水，美化城市环境。实施治乱塑景工程，集中整治重点路口、偏僻街区、老旧小区、低端市场，加快城隍庙市场搬迁；高标准、大力度整治城市出入口，打造靓丽整洁、畅通有序的城市迎宾通道。

(三)坚定不移地推进农业现代化和新型城镇化。农业现代化和新型城镇化是实现城乡一体化的关键所在，两者相辅相成、互为依托，必须整合资源、统筹推进。

大力发展现代农业。推进整建制粮食高产和玉米千亩吨粮创建，深入实施高产创建以奖代补支持政策，确保主要农产品稳定增产。整合土地整理、小农水建设等政策性资金，集中连片抓好26万亩高标准农田建设。积极构建现代农业经营体系，加快农村集体产权制度改革，培育新型经营主体，稳妥推进土地流转，新增土地流转面积50万亩，新发展家庭农场150个、农民合作社500家。加快国家农业产业化示范基地建设，鼓励工商资本进入农业领域，发挥供销社基层网络优势，围绕蔬菜、畜牧、林果、花卉等特色产业，新建优质安全蔬菜产业园区20个，发展标准化规模养殖场200家以上。健全农产品质量安全、农业生态安全和动植物疫情防控体系。大力发展农产品精深加工和仓储物流产业。推广先进适用技术，提升农机装备水平。促进农民职业化，加快培养新型农业从业队伍。

积极推进新农村建设。坚持农村社区、产业园区、基础设施、公共服务同步推进，优化村庄布局，改善生产生活条件。 “两区同建”将充分利用新型农村城镇化融资试点和土地增减挂钩政策，优先安排城中村、城边村和乡镇驻地，开工建设农村社区40个，续建158个，打造精品社区30个，入住10万户；每个县(市、区)建成3—5处万亩以上的大型现代农业园区，每个乡镇建成2处500亩以上的特色农业园区。用足用好国家支农政策，引导更多资金投向农村道路、环境整治和公共服务体系，让农村成为安居乐业的美丽家园。

全面提升城镇承载能力。着力做大做强县城，争取陵县撤县设区，引导武城、平原、宁津加速融入中心城区，支持其他县城扩容提质。按照特色小城市的标准，积极推动扩权强镇，加快15个示范镇建设，搞好工业、商贸、居住功能分区，打造县域次中心。按照上级统一部署，积极推进户籍制度和社会管理改革，逐步实现社保、教育、医疗、住房等基本公共服务城镇常住人口全覆盖。

科学搭建城乡路网框架。按照对内大循环、对外大开放的要求，加快推进德商高速、石济客运专线和济齐黄河大桥建设，确保济乐高速、德大铁路、邯济铁路竣工运营，做好聊城—德州—黄骅铁路、济乐高速临邑连接线前期工作，打通德州市与外围大城市、大港口、大机场的快捷通道。加快推进S318改建工程，争取S101改线和德商、京台、京沪高速连接线项目列入省级规划，完善提升中心城区到县城快速交通联系，形成市与县1小时交通圈。抓住全省村级公路网化示范县建设的机遇，继续调整优化乡村路网体系，新建改造农村公路1300公里，提高县城与乡镇之间的畅通能力，尽快建成外通内畅、贯穿城乡的现代交通体系。

(四)坚定不移地构建宜居宜业生态德州。推进绿色、循环、低碳发展，促进生产空间集约高效、生活空间宜居适度、生态空间舒适优美，让德州天更蓝、地更绿、水更清。

继续推进城乡大绿化、大水网建设。抓好沿河绿道、生态廊道、环城绿带、农田林网建设，全年新植树木3000万株、造林25万亩，森林覆盖率力争达到35%。巩固中心城区“创城”成果，完善城市绿地系统，加快主干道路、公共绿地、街头绿地、单位庭院和住宅小区绿化建设，改造提升大型公园绿地20处，完成城区道路绿化50万平方米，形成绿带环城、绿道穿城、绿意满城的绿色德州。按照水系贯通、并连成网、循环流动、水清岸绿的目标，加快南水北调续建配套、中小河流治理、灌区改造等重点工程建设，提升城乡供水一体化水平。完善“治用保”防控体系，抓好污水、垃圾处理设施的建设、改造和运营，实施“千亩湿地行动”，确保主要河流断面水质稳定达到地表水Ⅴ类标准，实现海河迎查夺冠目标。严格水资源管理，保护城乡饮用水源，推进水循环利用，创建水生态文明城市。

坚决治理大气污染。强力实施蓝天行动计划，推进大气污染点源达标工程建设，抓好华能德州电厂、恒源石化、中联大坝、凯元热电等重点燃煤企业脱硫脱硝设施改造，实施小机组燃煤锅炉脱硫再提高工程。持续开展工地扬尘、汽车尾气、餐饮油烟等综合整治，加大“黄标车”淘汰力度，加强PM2.5监测治理，切实减少灰霾污染，努力增加“蓝天白云、繁星闪烁”天数。

扎实推动绿色集约发展。实施严格的环境准入标准，严禁新上不符合环保准入条件的项目，加快淘汰落后产能。实施节能科技提效工程和重点用能企业分级管理，推进建筑、交通、公共机构和居民生活等领域的节能，控制能源消费总量。严守耕地保护红线，严格土地用途管制，规范城乡建设用地市场，盘活存量建设用地，严查违法违规用地，提高土地节约集约利用水平。积极发展循环经济和环保产业，推进清洁生产、循环综合利用，开展绿色单位创建活动，加快形成节约资源能源和保护生态环境的产业结构、增长方式、消费模式。

(五)坚定不移地推进幸福德州建设。继续加大民生投入，落实惠民政策，在群众普遍关注的教育、医疗、文化、就业、社保、交通、市政、居住、弱势群体救助等领域，集中力量办好一批年度民生实事，促进公共服务均等化，让全市人民切身感受到民生在改善、福利在增加。

均衡化发展社会事业。立足于解决公共资源配置城乡不平衡、中心城区东西部不协调两大矛盾，乡村以增加供给总量为主，城市以优化布局结构为主。全面实施教育优先发展战略，继续推进农村中小学校舍标准化建设，新建、改扩建中小学80所、幼儿园100所，新增中小学学位3万个，优先补充乡村教师力量，提高师资水平。城区采取新建扩建、提升改造、整体搬迁、引进名校等方式，推动优质教育资源均衡布局，缓解人口密集区入学难、班额大问题；抓好德州高级师范学校、明诚学校建设和实验中学扩建。全面落实省级职业教育创新发展试验区工作任务，推进职业教育公共实训中心和德州职业技术学院二期工程建设。深化医药卫生体制改革，扩大县级公立医院综合改革试点，探索实施医联体建设；加快市人民医院新门诊楼建设，规划建设东部医疗城，促进医疗资源与城市发展相协调；完善公共卫生服务机制，推动卫生信息化、重点学科建设上水平；鼓励社会资本办医，发展医养结合健康服务业。加快构建现代公共文化服务体系，完善文化服务设施，组织文化惠民活动，实施文保工程和运河保护与展示工程。广泛开展全民健身活动，积极备战参赛省23届运动会。

广覆盖完善社保体系。加强就业创业培训，设立创业扶持基金，开工建设市人力资源市场，新增城镇就业6.5万人、农村劳动力转移就业8万人。加大各类保险扩面征缴力度，完成城镇居民基本医疗保险和新型农村合作医疗并轨，基本实现城乡养老保险、医疗保险人员全覆盖。不断提高困难群体保障水平，城市低保标准每人每月提高40元，农村低保每人每年提高200元，五保集中和分散供养标准每人每年分别提高到4000元、3000元。加快推进以居家为基础、社区为依托、机构为支撑的社会养老服务体系建设，新建改造养老床位9000张。建设市残疾人康复中心，发展社区康复机构。推动公租房、廉租房并轨，开工保障性安居工程26288套，缓解低收入群体住房困难。

深层次推进社会治理。创新预防和化解社会矛盾体制，健全重大决策社会风险评估机制，加强信访工作，开工建设市群众来访中心，使群众问题能反映、矛盾能化解、权益有保障。深入开展平安创建活动，加强社会管理综合治理，创新推行网格化管理模式。建立隐患排查治理和安全防控体系，坚决遏制重特大安全生产事故。健全应急管理机制，提升突发事件应对和处置能力。完成食品药品监管体制改革，严格全过程监管，保障群众饮食用药安全。高度重视国防动员、双拥共建和民兵预备役工作，依法保障老年人、妇女、儿童、青少年权益，认真做好民族宗教、外事侨务、防震减灾、档案、史志等工作。

(六)坚定不移地建设人民满意政府。以人民需求为导向，以人民满意为标准，开拓创新，积极作为，不断提高施政能力和服务水平。

努力建设听民声、顺民意的政府。坚持开门决策，多方汇集民智，重大决策前深入调查研究，对社会关注度高、影响面大的事项，根据需要组织专家论证、群众听证、媒体公开讨论，真正使政府决策建立在广泛的民意基础上。认真开展党的群众路线教育实践活动，建立联系和服务群众长效机制，接地气，办实事，解难题。大力提升“12345”市民热线服务效能，提高问题办结率和群众满意度。定期对群众反映集中的热点难点问题，系统梳理，专题研究，拿出办法，妥善解决。

努力建设服务优、效能高的政府。进一步压减审批事项，缩短审批时限，对已取消下放的，加大督查力度，防止反弹；全面清理收费项目，公开收费标准，坚决整治高收费、乱收费；规范中介机构，推行中介服务市场化，大力优化营商环境。加大政府向社会购买服务力度。积极推进电子政务和政务公开，完善政府信息发布制度。开展“庸懒散”专项治理，着力解决不作为、慢作为、乱作为问题。

努力建设讲规则、守诚信的政府。完善规章制度，严格依法行政，重大决策、重要文件、重要合同要进行集体研究、合法性审查，定期清理规范性文件和行政处罚事项，规范行政执法行为。实施全面规范、公开透明的预算制度，强化预算硬约束，所有政府投资项目实行投资评审和绩效管理。加强政务服务中心和公共资源交易市场的运行监管，做到阳光操作、公平公正。严格政府性债务管理，加强源头规范，完善预警机制和动态监控办法，防止出现系统性、区域性政府债务风险。抓好政府诚信建设，以政府诚信带动全社会诚信。

努力建设重监督、严自律的政府。自觉接受人大、政协、群众以及媒体监督，广泛听取民主党派、工商联、人民团体和社会各界的意见，认真做好人大代表建议和政协提案办理工作。深入实施廉政效能管理工程，健全完善惩治和预防腐败体系，防控廉政风险，加强对重点岗位、重点资金和重点项目的审计监督，严厉查处各种违法违纪案件。坚决反对“四风”，厉行勤俭节约，压缩“三公”经费，严控政府性楼堂馆所建设，以勤政为民、清正廉洁的形象赢得人民群众支持和拥护。

各位代表!我们的所有工作、所有努力、所有奋斗，归根结底都是为了全市人民生活得更加幸福、更加自豪、更有尊严。德州正处在跨越发展的关键时期，责任重大，使命光荣。让我们在中共德州市委的坚强领导下，紧紧依靠全市人民，万众一心，攻坚克难，创新实干，圆满完成全年目标任务，为推动跨越发展、建设幸福德州而努力奋斗!
